# *Ganoderma lucidum* Protects Dopaminergic Neuron Degeneration through Inhibition of Microglial Activation

**DOI:** 10.1093/ecam/nep075

**Published:** 2011-06-18

**Authors:** Ruiping Zhang, Shengli Xu, Yanning Cai, Ming Zhou, Xiaohong Zuo, Piu Chan

**Affiliations:** Beijing Institute of Geriatrics and Department of Neurobiology and Neurology, Key Laboratory for Neurodegenerative Diseases of Ministry of Education, Xuanwu Hospital of Capital Medical University, #45 Changchun Street, Beijing 100053, China

## Abstract

Abundant evidence has suggested that neuroinflammation participates in the pathogenesis of Parkinson's disease (PD). The emerging evidence has supported that microglia may play key roles in the progressive neurodegeneration in PD and might be a promising therapeutic target. *Ganoderma lucidum* (GL), a traditional Chinese medicinal herb, has been shown potential neuroprotective effects in our clinical trials that make us to speculate that it might possess potent anti-inflammatory and immunomodulating properties. To test this hypothesis, we investigated the potential neuroprotective effect of GL and possible underlying mechanism of action through protecting microglial activation using co-cultures of dopaminergic neurons and microglia. The microglia is activated by LPS and MPP^+^-treated MES 23.5 cell membranes. Meanwhile, GL extracts significantly prevent the production of microglia-derived proinflammatory and cytotoxic factors [nitric oxide, tumor necrosis factor-**α** (TNF-**α**), interlukin 1**β** (IL-1**β**)] in a dose-dependent manner and down-regulate the TNF-**α** and IL-1**β** expressions on mRNA level as well. In conclusion, our results support that GL may be a promising agent for the treatment of PD through anti-inflammation.

## 1. Introduction

Parkinson's disease (PD) is a common neurodegenerative disease, clinically characterized by slowed movement, rigidity, rest tremor and disturbances in balance [1]. With the progression of the disease, many patients develop non-motor symptoms, including anxiety, depression, constipation and dementia. Although there are drugs that can alleviate PD symptoms, chronic use of these drugs is not effective in deterring the progression of PD and has been associated with debilitating side effects. It is therefore of great interest to develop neuroprotective therapies aimed at slowing or even halting the degenerative progression [2]. However, the development of effective neuroprotective therapies is impeded by our limited knowledge of the pathogenesis of PD.

The etiology and pathogenesis responsible for the neuronal degeneration in PD remains unknown. Several line of evidence supported that glia activation and inflammatory processes are involved in the cascade of events leading to progressive degeneration [3, 4]. Numerous activated microglia are present in the vicinity of degenerating neurons in the substantia nigra of patients with PD [5]. In the mature brain, microglia typically exists in a resting state characterized by ramified morphology, and monitors the brain environment. In response to abnormal stimuli, such as environment toxins, neurotoxins, microglia becomes activated and can induce significant and highly detrimental neurotoxic effects by the excess production of a large array of cytotoxic factors such as nitric oxide (NO), tumor necrosis factor-*α* (TNF-*α*), interleukin 1*β* (IL-1*β*) and superoxide, and so forth [3, 5–7].

*Ganoderma lucidum* (GL) is widely used as an alternative medicine remedy to promote health for 1000 years in China. Studies have indicated that components of GL extract have a wide range of pharmacological actions including immunomodulation, suppressing inflammation, promoting mitochondria energy production and scavenging free radicals [8–11]. Previous work, shows that GL extracts could prevent neuronal loss after cerebral ischemia [12]. But it is not known whether GL could protect against dopaminergic neuron degeneration and attenuate the inflammatory responses of microglial cells to exogenous or endogenous stimulus. We set out to answer these questions in the current study.

## 2. Methods

### 2.1. Materials

GL extracts were generously provided by PuraPharm Corporation (Guangxi, China) with a lot number: 070106. The extracts were prepared from the fruiting body with methanol and low temperature extraction technology. The major components consisted of mainly polysaccharide triterpenes and ergosterol. The GL extracts used were defined by a content of polysaccharides and ergosterin. According to the calculation, the yield of polysaccharide was 0.6% (w/w) in terms of the fruiting body of Ganoderma, and ergosterol was 0.35%. GL was resolved in phosphate-buffered saline. Cell culture reagents were obtained from Gibco (Grand Island, NY, USA) and [^3^H] dopamine (DA) was purchased from PerkinElmer Life Science (Boston, MA, USA). Lipopolysaccharides and Griess reagent were purchased from Sigma (St Louis, MO, USA). The monoclonal antibody against rat CD11b (OX-42) was obtained from Serotec (Oxford, UK). Diaclone (Besancon, France) supplied Rat TNF-*α* detection ELISA kits, while superoxide Assay Kit-WST and rat IL-1*β* ELISA kits were obtained from Dojindo (Kyushu, Japan) and IBL (Gunma, Japan), respectively. The real-time PCR reagents were provided by Takara (Tokyo, Japan).

### 2.2. Cultures of Microglia and MES 23.5 Cells

Microglia were isolated and purified from brains of 12–24-h-old Wistar rats supplied by Laboratory Animal Center [13]. The research was conducted in accordance with the Declaration of Helsinki and with the Guide for Care and Use of Laboratory Animals as adopted and promulgated by the United National Institutes of Health. All experimental protocols were approved by the Review Committee for the Use of Human or Animal Subjects of Capital Medical University. Briefly, after brains were dissected and the meninges removed, the tissues were minced and digested with trypsin (0.25% trypsin-EDTA in 0.1-M phosphate buffer) for 20 min at 37°C, triturated with a fire-polished Pasteur pipette and filtered through a 200-*μ*M nylon cell strainer. After centrifugation for 5 min at 121 g, the tissue were suspended into DMEM containing 10% fetal bovine serum (FBS), and seeded in 75-cm^2^ flasks at a density of 5 × 10^5^/ml cells per flask. Two weeks after the seeding, the flasks were shaken at 180 rpm for 4 h, and the floating cells were collected and centrifuged for 5 min at 800 rpm, the cells were resuspended and plated to 96-well plates for further experimental treatment.

The dopaminergic cell line MES 23.5 was a gift from Prof. Wei-dong Le, Department of Neurology, Baylor College of Medicine, Houston. The MES 23.5 cells were derived from somatic cell fusion of rat embryonic mesencephalic cells with murine N18TG2 neuroblastoma cells [14]. MES 23.5 cells display many prosperities of developing neurons of the SN zona compacta and offer several advantages for such initial studies, including greater homogeneity than primary cultures and susceptibility to both free-radical-mediated cytotoxicity and calcium-dependent cell death. MES 23.5 cells were seeded on polylysine-precoated 24-well plates at a density of 10^4^ cells/cm^2^ and maintained in DMEM with Sato's components at 37°C in a 95% air/5% CO_2_ humidified atmosphere incubator. Some of the cultured MES 23.5 cells were co-cultured with microglia.

To study the interaction of reactive microglia with MES 23.5 cells, microglia and MES 23.5 cells were co-cultured in 24-well culture plates. Briefly, the purified microglia were plated at a density of 1 × 10^4^/well 1 day before addition of MES 23.5 cells at a ratio of 2 : 1 (MES 23.5 to microglia). The co-cultures were maintained in Sato's conditioned medium containing 2% heat-inactivated FBS. The cultures of microglia or MES 23.5 cells alone or together were treated for 24 h with lipopolysaccharide (LPS, 0.25 *μ*g/ml) as a positive control, GL extracts (50–400 *μ*g/ml) or MES 23.5 cell membrane constituents (150 *μ*g/ml) [13].

### 2.3. Immunocytochemistry

Paraformaldehyde-fixed cell cultures were immunostained as described previously [15]. Microglia was stained with a monoclonal antibody OX-42. Briefly, cell cultures were treated for 15 min with 3% H_2_O_2_, then blocked with appropriate normal serum followed by incubation overnight at 4°C with a primary antibody diluted in antibody diluents [15]. After incubation with an appropriate biotinylated secondary antibody and then the ABC reagents, the bound complex was visualized by color development with 3,3′-diaminobenzidine (DAB). Images were recorded with a Nikon inverted microscope.

### 2.4. Preparation of MES 23.5 Cell Membrane Fraction

After exposure to MPP^+^ 10 *μ*M for 24 h, the MES 23.5 cells were harvested in a buffer containing 0.25-M sucrose, 100-mM PBS, 1-mM MgCl_2_, 1-mM EDTA and 2-*μ*M protease inhibitor PMSF, and homogenized with a glass-teflon homogenizer [13]. Then the homogenate was centrifuged at 8000 g for 10 min at 4°C to remove the crude nuclear fractions. The supernatants were again centrifuged at 100 000 g for 60 min at 4°C. The precipitates were homogenized and suspended in culture medium and used as the neuronal membrane fractions.

### 2.5. High-Affinity [^3^H] DA Uptake Assay

Cells in each well were washed with 1 ml of Krebs-Ringer buffer (16-mM NaH_2_PO_4_, 16-mM Na_2_HPO_4_, 119-mM NaCl, 4.7-mM KCl, 1.8-mM CaCl_2_, 1.2-mM MgSO_4_, 1.3-mM EDTA and 5.6-mM glucose; pH 7.4). The cells were then incubated with 10-nM [^3^H]DA in Krebs-Ringer buffer (10 *μ*l/well) for 30 min at 37°C [15]. Non-specific uptake of dopamine was determined in parallel wells receiving both dopamine and 1-mM nomifensine (10 *μ*l/well), an inhibitor of neuronal high-affinity dopamine uptake. Afterward, the cells were washed three times with ice-cold Krebs-Ringer buffer (1 ml/well) and lysed with 1 N NaOH (0.5 ml/well). After mixing the lysate with 3 ml of scintillation fluid overnight, radioactivity was determined with Perkin Elmer 1450LSC Luminescence Counter (Waltham, USA). Specific uptake was determined by subtracting the non-specific counts for the total activity.

### 2.6. NO Assay

The production of NO was quantified by measuring the released NO metabolites (nitrates and nitrites) with Griess reagent [16]. After a 24 h exposure to LPS/cell fraction, the culture medium samples were collected and prepared cell-free by centrifugation. The medium was incubated with the same volume of Griess reagent at room temperature for 10 min before measuring absorbance at 540 nm in a LP-400 ELISA reader (Diagnostics Pasteur, Marne-la-Coquette, France) with appropriate standards.

### 2.7. TNF-*α*, IL-1*β* and Superoxide Assay

Samples were prepared similar to NO samples and the production of these factors were determined using rat TNF-*α* kit, rat IL-1*β* ELISA kit and superoxide Assay Kit-WST according to the manufacturer's instructions. Then, run the plate reader and conduct measurement at 450 nm.

### 2.8. RNA Isolation and Real-Time PCR

Total RNA was extracted from primary microglial cells using RNAprep Kit according to the manufacturer's specifications. RNA was primed with random 9-mers and converted into cDNA by reverse transcription (RT) using AMV reverse transcriptase by following the manufacturer's recommended protocol [17, 18]. The resulting cDNA was then subjected to real-time PCR with SYBR Premix Ex Taq containing a final concentration of 1× SYBR Green (Molecular Probes) and 0.2 *μ*M of the primer set of interest in a 20-*μ*l reaction. The PCR mixture was run in the DNA engine Opticon 2 (MJ research; Waltham, MA). After an initial 10 s 95°C denaturation step, the reaction was run through 35 cycles at 95°C for 5 s, 60°C for 30 s and 80°C for 1 s. Melting curve analysis was executed to ensure the resulting products from the reaction had equivalent and appropriate melting temperatures. The specific primers used are listed in Table 1 [17]. The quantification of target transcripts was based on a calibration curve. The “housekeeping” gene *β*-actin was targeted for an internal control gene. The test gene data were normalized by corresponding *β*-actin data.

### 2.9. Statistical Analysis

Data were expressed as the means ± SD. Statistical significance was assessed with an analysis of variance (ANOVA) followed by LSD post hoc test using SPSS 11.5. A value of *P* < .05 was considered to be statistically significant.

## 3. Results

### 3.1. Microglial Activation Induced by LPS and MPP^+^-Treated Dopaminergic Cell Membranes

**Figure 1:**
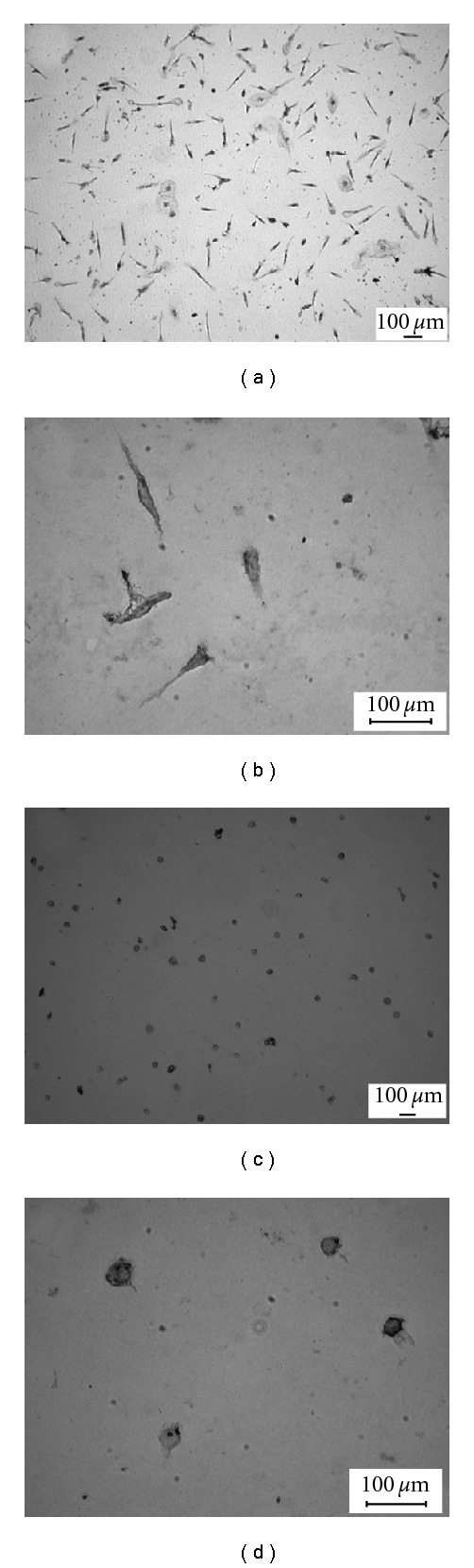
The morphology of rat microglia cells labeled with OX-42. Rat microglia were incubated for 24 h with vehicle ((a) 100x; (b) 400x), LPS 0.25 *μ*g/ml ((c) 100x; (d) 400x). Microglia were transformed into an amoeboid morphology after treated with LPS. Scale bar represents 100 *μ*m.

To establish models of microglia activation in neurodegeneration, LPS and MPP^+^-treated dopaminergic cell membranes were used as stimuli in either microglia culture or dopaminergic neuron (MES 23.5 cell line) and microglia co-cultures. Microglia cells were visualized by staining for the CR3 complement receptor using monoclonal antibody OX-42. The purity of microglia cultures is *∼*95%. The quiescent microglia displayed either a ramified shapes or bipolar or multipolar processes (Figures 1(a) and 1(b)) [19, 20]. The activated microglia displayed amoeboid morphology (Figures 1(c) and 1(d)). 

**Figure 2:**
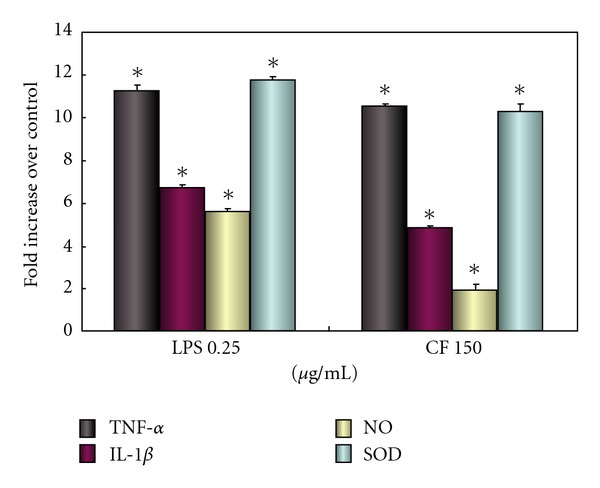
LPS and CF increase cytokines through activating microglia. Microglial activation was determined by measuring the levels of TNF-*α*, IL-1*β*, NO and superoxide in cells after exposure to LPS (0.25 *μ*g/ml) and CF (150 *μ*g/ml). The levels of TNF-*α*, IL-1*β*, NO and superoxide in vehicle controls are 106.55 pg/ml, 119.09 pg/ml, 0.6 *μ*M and 5.22 U/ml, respectively. Levels were expressed as fold increase as compared to concentrations of the control. All cytokines were increased significantly (*P < .05).

Of the numerous neurotoxic factors, NO, TNF-*α*, IL-1*β* and superoxide may be major mediators of dopaminergic neurodegeneration elicited by microglial activation. We first characterized the LPS-induced microglial activation by measuring the levels of TNF-*α* and IL-1*β*, two well-documented cytokines reflecting microglial activation, and the levels of several reactive oxygen species (ROS, NO and superoxide) released from activated microglia. Unstimulated microglia produce very low amounts of any cytokine which is negligible. After exposed to LPS (0.25 *μ*g/ml), the levels of TNF-*α* and IL-1*β* were increased by 6–11-fold, and the levels of NO and superoxide were elevated up to 5–11-fold in the microglia culture media (Figure 2). Because MES 23.5 cells activated microglia only after MPP^+^ treatment [19], we examined the activation effects of MES 23.5 cell membrane fractions (CF) treated with MPP^+^. Incubation with MPP^+^-treated cell membrane fraction (150 *μ*g/ml), TNF-*α* and IL-1*β* production was significantly increased by 4–10-fold (Figure 2). The levels of NO and superoxide from the MPP^+^ membrane fraction-treated microglial culture media were increased by 2–10-fold (Figure 2). Crude membrane without MPP^+^ or treated with GL only had minimal activating effects as compared with MPP^+^ membrane fraction (data not presented). 

### 3.2. GL Prevents the Production of Proinflammatory Factors and ROS Derived from Microglia

**Figure 3:**
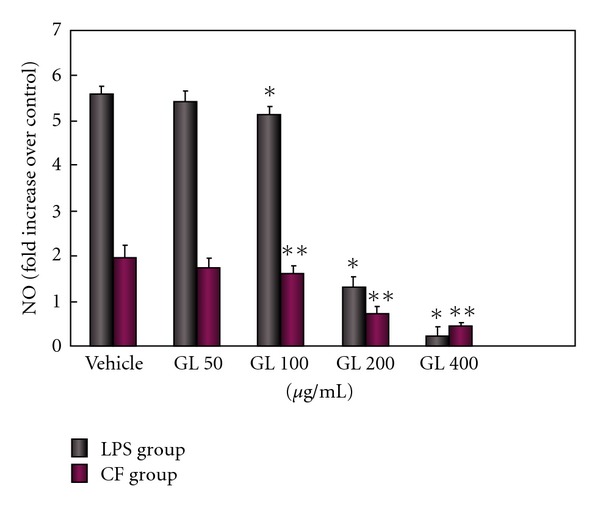
GL protects against LPS or CF induced production of NO in a dose-dependent fashion. Cultures were treated with GL at indicated concentration 30 min prior to exposure with 0.25 *μ*g/ml LPS or 150 *μ*g/ml CF. Culture supernatants were collected and assayed for NO. Data are expressed as fold increase of control group and presented as means ± SD of two experiments performed in triplicate. *P < .01 compared with LPS only treated cultures and **P < .05 compared with CF only treated cultures.

**Figure 5:**
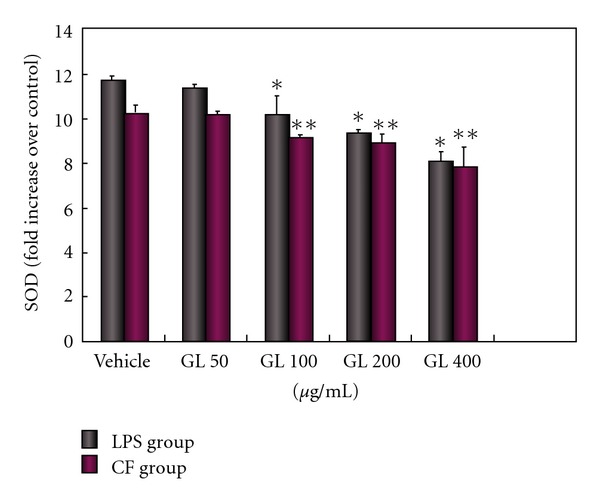
GL protects against LPS or CF induced production of superoxide in a dose-dependent fashion. Cultures were treated with GL at indicated concentration 30 min prior to exposure with 0.25 *μ*g/ml LPS or 150 *μ*g/ml CF. Superoxide generation was measured with the SOD assay kit-WST. Data are expressed as fold increase of control group and presented as means ± SD of two experiments performed in triplicate. *P < .001 compared with LPS only treated cultures and **P < .05 compared with CF only treated cultures.

**Figure 6:**
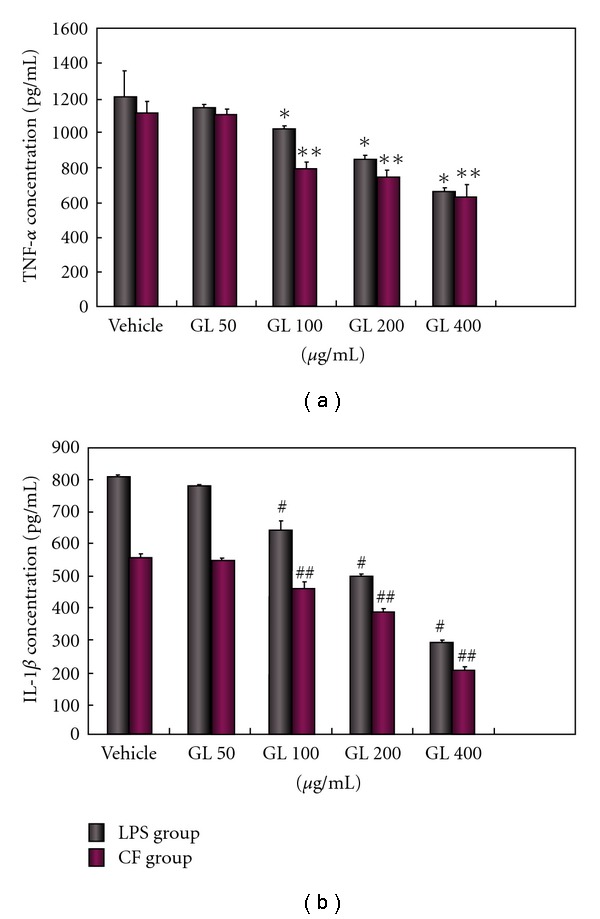
GL protects against LPS or CF induced production of TNF-*α* (a) and IL-1*β* (b) in a dose-dependent fashion. Cultures were treated with GL at indicated concentration 30 min prior to exposure with 0.25 *μ*g/ml LPS or 150 *μ*g/ml CF. TNF-*α* and IL-1*β* levels were determined as described in Methods section. Data are expressed as means ± SD of two experiments performed in triplicate. *P < .05 and **P < .001 compared with LPS and CF only treated cultures for TNF-*α*. ^#^ *P* < .001 and ^##^ *P* < .001 compared with LPS and CF only treated cultures for IL-1*β*, respectively.

Microglia can produce cytokines as a consequence of activation [21–23]. To elucidate the underlying mechanism of the neuroprotective activity of GL, we investigated the effect of GL on levels of microglia-derived inflammatory cytokines and ROS. Microglial cell cultures were pretreated with different dosages (50–400 *μ*g/ml) of GL for 30 min followed by exposure to LPS or CF treated with MPP^+^. As shown in Figures 3 and 5, low dose (50 *μ*g/ml) of GL had minimal inhibiting effects, while pretreatment with higher dose of GL (100–400 *μ*g/ml) potently reduced the increase of NO and SOD caused by LPS or CF in a concentration-dependent fashion. The higher dose of 400 *μ*g/ml almost had a complete prevention of the production of NO. At the equivalent concentration, GL also significantly decreased the release of TNF-*α* and IL-1*β* after LPS and CF treated with MPP^+^ (Figure 6).

### 3.3. GL Protects against MPP^+^-Induced Dopaminergic Neurodegeneration in the Presence and Absence of Microglia

**Figure 7:**
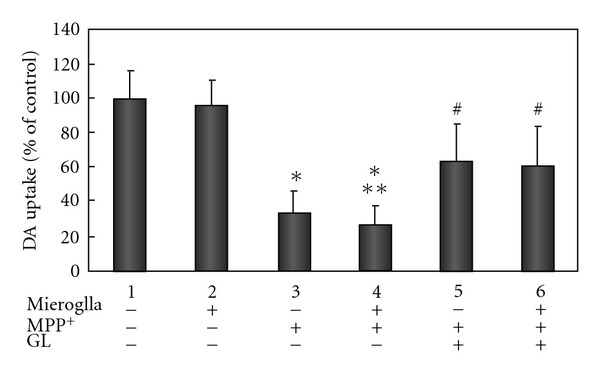
GL protects against MPP^+^-induced reduction of [^3^H] DA uptake in MES 23.5 cell cultures with or without microglia. The cultures were treated with vehicle or 100 *μ*M MPP^+^, and 400 *μ*g/ml GL. GL was given 30 min before the MPP^+^ exposure. Uptake of [^3^H] DA was assessed as described in Methods section. Data were generated from cell samples in duplicated experiments, and are expressed as percent of the vehicle group. *P < .001 compared with corresponding MES 23.5 in the absence or presence of microglia cultures without exposure to MPP^+^; **P < .05 compared with the MPP^+^-treated MES 23.5 cultures without microglia; ^#^ *P* < .001 compared with corresponding MES23.5 in the absence or presence of microglia cultures without exposure to GL.

To assess the inflammation mediated neurotoxicy, dopaminergic MES 23.5 neurons were exposed to 100 *μ*M MPP^+^ or 0.25 *μ*g/ml LPS in the absence or presence of microglia co-culture for 24 h, and neurotoxicity was assessed using [^3^H] DA uptake assay. Exposure to MPP^+^ leaded to a significant decrease in [^3^H] DA uptake by about 66% for MES 23.5 neurons alone, while about 74% decrease was noted for MES 23.5 and microglia co-cultures (Figure 7). Pretreatment with 400 *μ*g/ml GL significantly protected MPP^+^-induced reduction of [^3^H] DA uptake, which only decreased by *∼*35% and 38%, respectively, in the absence and presence of microglia co-cultures. 

### 3.4. GL Protects against LPS-Induced Dopaminergic Degeneration in the Presence of Microglia

**Figure 4:**
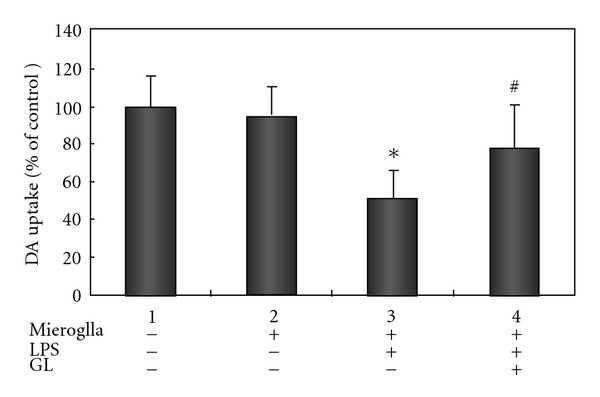
GL protects against LPS-induced reduction of [^3^H] DA uptake in MES 23.5 cell cultures with or without microglia. The cultures were treated with vehicle or 0.25 *μ*g/ml LPS, and 400 *μ*g/ml GL. GL was given 30 min before the LPS exposure. Uptake of [^3^H] DA was assessed as described in Methods section. Data were generated from cell samples in duplicated experiments, and are expressed as percent of the vehicle group. *P < .05 compared with corresponding MES 23.5 in the absence or presence of microglia cultures without exposure to LPS; ^#^ *P* < .01 compared with corresponding MES 23.5 and microglia co-cultures without exposure to GL.

When the neuron-microglia co-cultures were exposed to 0.25 *μ*g/ml LPS for 24 h, [^3^H] DA uptake was significantly reduced by *∼*50% as compared to co-cultures (Figure 4). Pretreatment of co-cultures with 400 *μ*g/ml GL also significantly attenuated LPS-induced decrease in [^3^H] DA uptake (22% loss with GL versus 50% loss without GL). 

### 3.5. GL Inhibits the Increased Expression of TNF-*α* and IL-1*β* mRNA by LPS and MPP^+^-Treated Membrane

**Figure 8:**
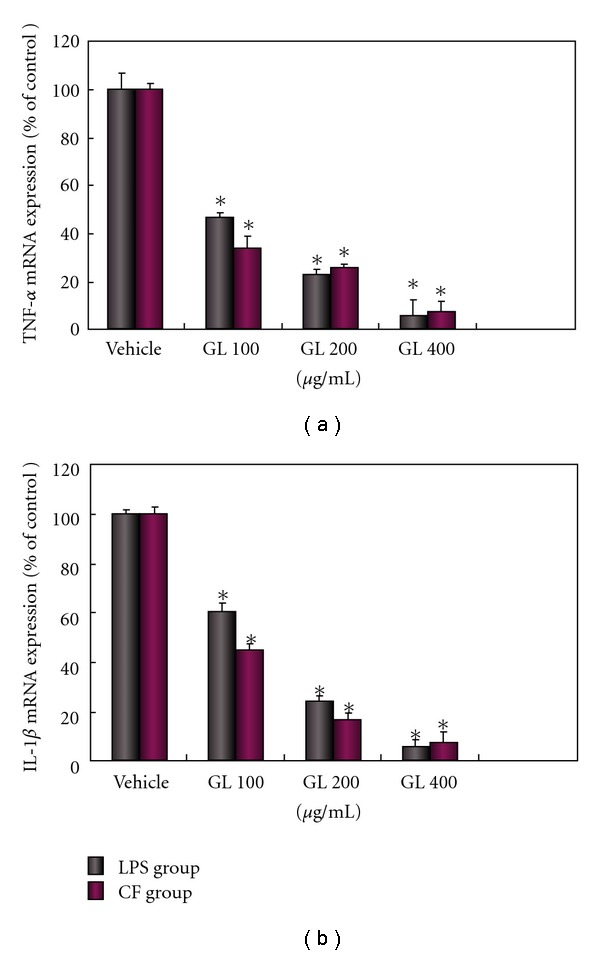
GL protects against LPS or CF induced overexpression of mRNA levels of TNF-*α* (a) and IL-1*β* (b) in a dose-dependent fashion. Cultures were treated with GL at indicated concentration 30 min prior to exposure with 0.25 *μ*g/ml LPS or 150 *μ*g/ml CF. Total RNA was extracted and then subjected to real-time PCR as described in Methods section. Data are expressed as percentage of the control group (LPS or CF only treated group, respectively) calculated from the average threshold cycle values and presented as the mean ± SD. Independent RNA preparations from different sets of cultures were prepared and determinations were performed in triplicate from the RNA samples of a set of experiment. *P < .05 compared with LPS or CF only treated cultures, respectively.

Synthesis of all these proinflammatory factors is controlled at several levels. Whereas post-transcriptional, translational and post-translational mechanisms play important roles, gene transcription appears to be the primary regulatory site. The levels of TNF-*α* and IL-1*β* mRNA expression were barely detectable in control cells but were significantly increased by LPS and CF. Pretreatment of 100–400 *μ*g/ml GL inhibited their expression in a dose-dependent manner. The higher dose of 400 *μ*g/ml GL provided a 90% protection (Figure 8). 

## 4. Discussion

In the present study, we have demonstrated that GL effectively protects dopaminergic neurons against inflammatory damage induced by microglial activation after exposure to MPP^+^ and LPS. The underlying mechanism appears to be related to the ability of GL to protect against the production of microglia-derived toxic factors (NO, TNF-*α*, IL-1*β* and superoxide). This is supported by the observation that GL markedly down-regulate the mRNA expression of TNF-*α* and IL-1*β*.

PD pathologically is characterized by progressive nigral cell degeneration which is accompanied by microglial activation. Although it is suggested that environmental or endogenous toxins may lead to neuronal death, the mechanism underlying microglia activation remains unknown. It has been shown that LPS and neurotoxins can activate microglia and cause progressive neurodegeneration which can be prevented by inhibiting expression of proinflammatory factors [24]. Increasing evidence has linked chronic inflammation to a number of neurodegenerative diseases, including PD.

**Figure 9:**
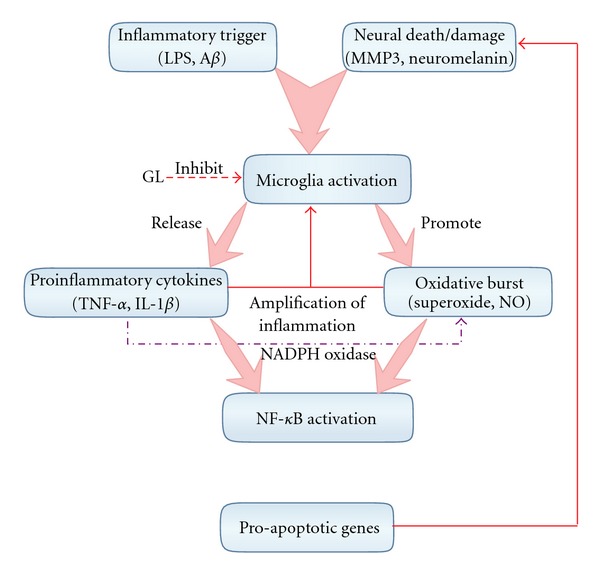
The molecular mechanisms between microglial activation and neuron death. Microglia can be activated by inflammatory trigger, such as LPS and other toxins and consequently produce proinflammatory factors and cytokines which on one side can cause auto-implication of ROS, NO and superoxide radicals to form highly oxidizing peroxynitrite species and also activate other resting microglia. TNF-dependent microglia activation in the SN creates an environment of oxidative stress through activation of NADPH oxidase. IL-1*β* can disrupt the blood brain barrier and facilitate the infiltration of leukocytes into CNS. All these factors can activate NF-*κ*B, which can up-regulate pro-apoptotic genes leading to neuronal death. These events form a vicious circle leading to progressive neuronal degeneration. GL can inhibit the generation of microglia-derived toxic factors through its anti-inflammatory effect.

Microglia, the resident innate immune cells of central nervous system, play major role in the neuroinflammatory process. Microglia can be activated and cause neurotoxicity through two mechanisms [20]. First, microglia can initiate neuron damage by recognizing inflammatory trigger, such as LPS and other toxins [24], becoming activated and producing neurotoxic proinflammatory factors and cytokines. Consequently, these factors can deplete the antioxidant of DA neurons, impair mitochondrial function, inhibit the re-uptake of glutamate [25], and initiate CNS tissue damage [26]. In addition, cytokines such as TNF-*α* can activate other resting microglia, potentiating inflammatory response that lead to auto-implication of ROS, NO and superoxide radicals to form highly oxidizing peroxynitrite species [6, 27]. TNF-dependent microglia activation in the SN creates an environment of oxidative stress through activation of NADPH oxidase [28]. IL-1*β* has been shown to be involved in the development of CNS inflammation through the disruption of the blood-brain barrier which facilitates the infiltration of leukocytes into CNS [23, 24]. NO is membrane permeable, excessive accumulation of NO could react with superoxide to form peroxynitrite which capable of attacking and modifying proteins, lipids and DNA as well as depleting antioxidant defenses [25, 26]. Much of the microglial-derived ROS such as superoxide cannot efficiently traverse cellular membranes, making it unlikely that these extracellular ROS gain excess to dopaminergic neurons and trigger intra-neuronal toxic events, however, superoxide can rapidly react with NO in the extracellular space to form a more stable oxidant, which can readily cross cell membranes and damage intracellular components in neighboring neurons [27]. All these factors can activate a key transcription factor, NF-*κ*B, which can up-regulate pro-apoptotic genes leading to neuronal death [29, 30]. Second, microglia can become overactivated in response to neuronal damage, which is then toxic to neighboring neurons [31, 32], resulting in a perpetuating cycle of neuron death. Several studies reveal that damaged DA neurons release matrix metalloproteinase 3 (MMP3) [33], *α*-synuclein [34] and neuromelanin [33, 35] that seem to activate microglia and are implicated in neuronal degeneration in PD. All these events form a vicious circle leading to progressive neuronal degeneration (Figure 9). 

In primary neuron-enriched or neuron-glia co-cultures derived from rat mesencephalon, rotenone-induced dopaminergic neurodegeneration was dependent on the presence of microglial cells. This was mediated by the production of superoxide from activated microglia [15]. The injection of LPS in the supra nigral area of rat brains resulted in microglial and astroglial proliferation and these reactive glial cells were important mediators of the subsequent neuronal death [36]. Furthermore, inhibition of glial cell activation in the mouse brain decreased MPTP-induced neurotoxicity by blocking the formation of iNOS and IL-1 [37]. Results of the current study suggest that the inhibition of microglia-derived toxic factors production may be a mechanism underlying the anti-inflammatory effect of GL. The putative mechanisms involved in the microglial activation and neuronal injury are illustrated in Figure 9.

GL is a natural herbal medicinal fungus that has been used in China since 100 AD. Clinically, GL has been used to lower cholesterol, improving memory and anti-aging. Experimentally, GL extracts have been demonstrated to possess immunomodulating, anti-inflammatory, anti-oxidative damage and antitumor activities, as well as neuroprotective effects [8, 10, 38]. Treatment with GL tended to increase mitogenic reactivity to phytohemagglutinin, counts of CD3, CD4, CD8 and CD56 lymphocytes, plasma concentrations of interleukin (IL)-2, IL-6 and interferon (IFN)-*γ*, and NK activity, whereas plasma concentrations of IL-1 and TNF-*α* were decreased in cancer patients [39]. Studies have also shown that administration of the GL daily once for 15 days, was significantly effective to enhance the Krebs cycle dehydrogenases, and mitochondrial electron transport chain complex IV activities in aged rats. The profound activity of the extract can be correlated to the significant antioxidant property of GL [40].

The most important pharmacologically active constituents of GL are polysaccharides and triterpenoids. Polysaccharides, especially *β*-d-glucans, have been known to possess anti-tumor effects through immunomodulation and anti-angiogenesis. In addition, polysaccharides have a protective effect against free radicals and reduce cell damage caused by mutagens. Triterpenoids have been reported to possess antioxidation, hepatoprotective, anti-hypertensive, hypocholesterolemic and anti-histaminic effects [41]. However, the active components that play the role in the current study need further investigation.

The current most effective symptomatic therapy for PD is levodopa administration, but the efficacy declines as the disease progresses. It is the current focus that the neuroprotective strategies to rescue nigral DA neurons from progressive death. Growing evidence have indicated that a range of Chinese herbs or herbal extracts such as green tea polyphenols [42], ginsenoside [43], ginkgo biloba [44] and polysaccharides have the potential to protect against degeneration of DA neurons and prevent symptoms [45]. In addition, studies have suggested that Chinese herbs or herbal extracts may promote neuronal survival and neurite growth, and facilitate functional recovery of brain injures due to their ability as the antioxidants, DA transporter inhibitor, monoamine oxidase inhibitor, free radical scavengers, chelators of harmful metal ions, modulating cell survival genes and signaling, anti-apoptosis activity, and even improving brain blood circulation [46]. New pharmaceutical strategies against PD will hopefully be discovered by understanding the various active entities and valuable combinations that contribute to the biological effects of Chinese herbs and herbal extracts.

In conclusion, contrary to current therapies such as DA replacement and adjuvant surgical therapy that relief most motoric symptoms, GL which is capable of inhibiting microglial activation, may prevent neurodegeneration and/or restore function in PD. In light of limited inventory effective drugs, further studies on GL as a potential drug for the treatment of PD are warranted. We anticipate that better treatments will arise from these studies in the near future.

## Funding

Ministry of Sciences and Technology of China grants (2004BA702B02, 2006CB500701, 2006AA02A408).

## Figures and Tables

**Table 1:** 

Sequence name	Abbreviation	Accession number	Forward primer	Reverse primer	Product size
Interleukin-1*β*	IL-1*β*	NM_008361	CCGTGGACCTTCCAGGATGA	GGGAACGTCACACACCAGCA	102 bp
Tumor necrosis factor *α*	TNF-*α*	NM_013693	CCACCACGCTCTTCTGTCTA	AGGGTCTGGGCCATAGAACT	116 bp

## References

[1] Parkinson J (2002). An essay on the shaking palsy. *The Journal of Neuropsychiatry and Clinical Neurosciences*.

[2] Dawson TM, Dawson VL (2002). Neuroprotective and neurorestorative strategies for Parkinson’s disease. *Nature Neuroscience*.

[3] Kreutzberg GW (1996). Microglia: a sensor for pathological events in the CNS. *Trends in Neurosciences*.

[4] Miller G (2005). Neuroscience. The dark side of glia. *Science*.

[5] McGeer PL, Itagaki S, Boyes BE, McGeer EG (1988). Reactive microglia are positive for HLA-DR in the substantia nigra of Parkinson’s and Alzheimer’s disease brains. *Neurology*.

[6] Tansey MG, McCoy MK, Frank-Cannon TC (2007). Neuroinflammatory mechanisms in Parkinson’s disease: potential environmental triggers, pathways, and targets for early therapeutic intervention. *Experimental Neurology*.

[7] Gao HM, Liu B, Zhang WQ, Hong JS (2003). Critical role of microglial NADPH oxidase-derived free radicals in the in vitro MPTP model of Parkinson’s disease. *FASEB Journal*.

[8] Lai KN, Chan LYY, Tang SCW, Leung JCK (2006). Ganoderma extract prevents albumin-induced oxidative damage and chemokines synthesis in cultured human proximal tubular epithelial cells. *Nephrology Dialysis Transplantation*.

[9] Lin J-M, Lin C-C, Chen M-F, Ujiie T, Takada A (1995). Radical scavenger and antihepatotoxic activity of Ganoderma formosanum, Ganoderma lucidum and Ganoderma neo-japonicum. *Journal of Ethnopharmacology*.

[10] Lindequist U, Niedermeyer THJ, Julich WD (2005). The pharmacological potential of mushrooms. *Evidence-Based Complementary and Alternative Medicine*.

[11] Yuhong YOU, Zhibin LIN (2002). Protective effects of Ganodema lucidum polysaccharides peptide on injury of macrophages induced by reactive oxygen species. *Acta Pharmacologica Sinica*.

[12] Xiu-shu W, De-ming D (2004). Protective effect of lingzhizongdai against nerve cell damnification. *Modern Medicine and Hygiene*.

[13] Le W-D, Rowe D, Xie W, Ortiz I, He Y, Appel SH (2001). Microglial activation and dopaminergic cell injury: an in vitro model relevant to Parkinson’s disease. *Journal of Neuroscience*.

[14] Crawford GD, Le W-D, Smith RG, Xie W-J, Stefani E, Appel SH (1992). A novel N18TG2 x mesencephalon cell hybrid expresses properties that suggest a dopaminergic cell line of substantia nigra origin. *Journal of Neuroscience*.

[15] Gao H-M, Hong J-S, Zhang W, Liu B (2002). Distinct role for microglia in rotenone-induced degeneration of dopaminergic neurons. *Journal of Neuroscience*.

[16] Mayer AM (1998). Therapeutic implications of microglia activation by lipopolysaccharide and reactive oxygen species generation in septic shock and central nervous system pathologies: a review. *Medicina (B Aires)*.

[17] Schell JB, Crane CA, Smith MF, Roberts MR (2007). Differential ex vivo nitric oxide production by acutely isolated neonatal and adult microglia. *Journal of Neuroimmunology*.

[18] Liu B, Du L, Hong JS (2000). Naloxone protects rat dopaminergic neurons against inflammatory damage through inhibition of microglia activation and superoxide generation. *Journal of Pharmacology and Experimental Therapeutics*.

[19] Hanisch U-K, Kettenmann H (2007). Microglia: active sensor and versatile effector cells in the normal and pathologic brain. *Nature Neuroscience*.

[20] Block ML, Zecca L, Hong J-S (2007). Microglia-mediated neurotoxicity: uncovering the molecular mechanisms. *Nature Reviews Neuroscience*.

[21] Fuxe KG, Tarakanov AO, Goncharova LB, Agnati LF (2008). A new road to neuroinflammation in Parkinson’s disease?. *Brain Research Reviews*.

[22] Rogove AD, Tsirka SE (1998). Neurotoxic responses by microglia elicited by excitotoxic injury in the mouse hippocampus. *Current Biology*.

[23] Wen LL, Chiu CT, Huang YN, Chang CF, Wang JY (2007). Rapid glia expression and release of proinflammatory cytokines in experimental Klebsiella pneumoniae meningoencephalitis. *Experimental Neurology*.

[24] Gao H-M, Jiang J, Wilson B, Zhang W, Hong J-S, Liu B (2002). Microglial activation-mediated delayed and progressive degeneration of rat nigral dopaminergic neurons: relevance to Parkinson’s disease. *Journal of Neurochemistry*.

[25] Persson M, Brantefjord M, Hansson E, Rönnbäck L (2005). Lipopolysaccharide increases microglial GLT-1 expression and glutamate uptake capacity in vitro by a mechanism dependent on TNF-*α*. *GLIA*.

[26] Taupin V, Renno T, Bourbonnière L, Peterson AC, Rodriguez M, Owens T (1997). Increased severity of experimental autoimmune encephalomyelitis, chronic macrophage/microglial reactivity, and demyelination in transgenic mice producing tumor necrosis factor-*α* in the central nervous system. *European Journal of Immunology*.

[27] Mosley RL, Benner EJ, Kadiu I (2006). Neuroinflammation, oxidative stress, and the pathogenesis of Parkinson’s disease. *Clinical Neuroscience Research*.

[28] Mander PK, Jekabsone A, Brown GC (2006). Microglia proliferation is regulated by hydrogen peroxide from NADPH oxidase. *Journal of Immunology*.

[29] Baeuerle PA, Henkel T (1994). Function and activation of NF-kappaB in the immune system. *Annual Review of Immunology*.

[30] Delhase M, Li N, Karin M, Ozes ON, Mayo LD, Gustin JA (2000). Kinase regulation in inflammatory response. Authors’ reply. *Nature*.

[31] Block ML, Hong J-S (2005). Microglia and inflammation-mediated neurodegeneration: multiple triggers with a common mechanism. *Progress in Neurobiology*.

[32] Teismann P, Tieu K, Cohen O (2003). Pathogenic role of glial cells in Parkinson’s disease. *Movement Disorders*.

[33] Kim YS, Kim SS, Cho JJ (2005). Matrix metalloproteinase-3: a novel signaling proteinase from apoptotic neuronal cells that activates microglia. *Journal of Neuroscience*.

[34] Zhang W, Wang T, Pei Z (2005). Aggregated *α*-synuclein activates microglia: a process leading to disease progression in Parkinson’s disease. *FASEB Journal*.

[35] Zecca L, Zucca FA, Wilms H, Sulzer D (2003). Neuromelanin of the substantia nigra: a neuronal black hole with protective and toxic characteristics. *Trends in Neurosciences*.

[36] Carvey PM, Chang Q, Lipton JW, Ling Z (2003). Prenatal exposure to the bacteriotoxin lipopolysaccharide leads to long-term losses of dopamine neurons in offspring: a potential, new model of Parkinson’s disease. *Frontiers in Bioscience*.

[37] Wu DC, Jackson-Lewis V, Vila M (2002). Blockade of microglial activation is neuroprotective in the 1-methyl-4-phenyl-1,2,3,6-tetrahydropyridine mouse model of Parkinson disease. *Journal of Neuroscience*.

[38] Yuen JWM, Gohel MDI (2005). Anticancer effects of Ganoderma lucidum: a review of scientific evidence. *Nutrition and Cancer*.

[39] Gao Y, Tang W, Dai X, Gao H, Chen G, Ye J (2005). Effects of water-soluble Ganoderma lucidum polysaccharides on the immune functions of patients with advanced lung cancer. *Journal of Medicinal Food*.

[40] Ajith TA, Sudheesh NP, Roshny D, Abishek G, Janardhanan KK (2009). Effect of Ganoderma lucidum on the activities of mitochondrial dehydrogenases and complex I and II of electron transport chain in the brain of aged rats. *Experimental Gerontology*.

[41] Boh B, Berovic M, Zhang J, Zhi-Bin L (2007). Ganoderma lucidum and its pharmaceutically active compounds. *Biotechnology Annual Review*.

[42] Pan T, Jankovic J, Le W (2003). Potential therapeutic properties of green tea polyphenols in Parkinson’s disease. *Drugs and Aging*.

[43] Van Kampen J, Robertson H, Hagg T, Drobitch R (2003). Neuroprotective actions of the ginseng extract G115 in two rodent models of Parkinson’s disease. *Experimental Neurology*.

[44] Kim MS, Lee JI, Lee WY, Kim SE (2004). Neuroprotective effect of Ginkgo biloba L. extract in a rat model of Parkinson’s disease. *Phytotherapy Research*.

[45] Ebadi M, Srinivasan SK, Baxi MD (1996). Oxidative stress and antioxidant therapy in Parkinson’s disease. *Progress in Neurobiology*.

[46] Radad K, Gille G, Moldzio R, Saito H, Ishige K, Rausch W-D (2004). Ginsenosides Rb1 and Rg1 effects on survival and neurite growth of MPP +-affected mesencephalic dopaminergic cells. *Journal of Neural Transmission*.

